# Latent growth curve modeling for the investigation of emotional factors in L2 in longitudinal studies: A conceptual review

**DOI:** 10.3389/fpsyg.2022.1005223

**Published:** 2022-09-08

**Authors:** Fang Zhang

**Affiliations:** School of Foreign Languages, Anhui University of Science and Technology, Huainan, China

**Keywords:** latent growth curve models, L2 affective variables, complex dynamics systems theory, temporal change, SLA

## Abstract

With the advent of Complex dynamic systems theory (CDST) in the field of second language question (SLA), the need for suitable CDST compatible methods for the investigation of temporal change in L2 affective variables has been felt more than before. One of the innovative methods for this purpose is latent growth curve modeling (LGCM), which has recently drawn the attention of SLA scholars. However, the application of this method is still a burgeoning demand in SLA. In response to this demand, the present study provides a review of the conceptualization, significance, and technical features of the implementation of LGCM. In doing so, this review suggests a number of practices *via* which LGCM has been introduced in SLA. Additionally, some practical implications are provided for SLA researchers to enhance their literacy of LGCM. Finally, future research suggestions for the progress of the use of this method in SLA are discussed.

## Introduction

The research domain in recent years has been largely influenced by the complex dynamic systems theory (CDST), as a new way of conceptualizing the reality of human life empirically. The emergence of CDST in the field of SLA has drawn the attention of researchers to the development of the variables in this field across time. In this paper, we discuss how CDST has also influenced language studies, and why there is a need for innovative research methodologies that can adequately reflect the dynamicity inherent to language development and classroom learning. Then, we move on to introduce latent growth curve modeling (LGCM) as an innovational method of quantitative research, which meets the requirements of studying the language learning process dynamically as embedded in a network of interactive forces in class. Finally, and most importantly, we review the existing body of L2 studies that have employed this approach to research so far. We will show that although these studies have been yet very few indeed, they have fruitful findings to contribute to the L2 theory and practice. The significance of these studies is reviewed and the potential implications they can have for the L2 teaching and learning domain. The nuanced procedures of these studies can guide future lines of research in L2 education. We will review how the innovative LGM research method, which lies within the greater structural equation modeling (SEM) framework can be effectively employed in empirical works of research into L2 development. We will review the proven advantages LGCM has had in L2 studies over traditional research methodologies, which make it the preferable means of tracing the detailed changes of teacher-or learner-related behaviors, personality traits, or psychological constructs in the immediate learning environment.

## Dynamicity implied in L2 studies

There are reasons why CDST is the best match for language studies. With its reaction against the deterministic predictability of dynamic phenomena and resistance against the conventional approaches to language or language development, as a process significantly affected by the time and place constraints, CDST suits the language-related body of research. The essentiality of exploring language learning constraints from a dynamic approach, taking into account the variability in intra-and inter-individual performance and the need to study the processes of change in the reality of classroom learning, was first raised by Larsen-Freeman (1997). Applied linguistics is marked by interdisciplinary and openness to external influences, which makes CDST a perfect match for studies in this domain. A complete overview of CDST in applied linguistics was published by Larsen-Freeman and Cameron (2008a), describing the self-organizing, interconnected and co-adaptive nature of language learning and viewing the language process as emerging always out of interactions.

In order to systematically study, the processes of change in language development and bring firm evidence for that, effective methodological processes are needed. As suggested, researchers in applied linguistics are expected to go beyond describing and theorizing changes in dynamic language systems and should do research to produce evidence for language development, in the light of the CDST. Yet, many researchers in language studies have hardly employed research methodologies that could adequately account for inter-and intra-individual differences and also the spatiotemporal constraints of language learning because of the ineffective and, better say, insufficient research methodologies they employed to study the processes of change in language development. We move on to introduce LGCM as an effective research methodology in quantitative and empirical language studies. Shortly afterward, the existing body of research (in L2 studies) which used this innovative research method is reviewed to see what valuable contributions they managed to make to the field of applied linguistics.

## Distinctive features of LGCM techniques

Applied linguistics research is fraught with conventional methods of analysis employed to follow and record variation in the human actor (most dominantly teacher and student) behavior or trait. Examples are regression analysis, comparison of mean scores, and repeated-measures analysis of variance. Nevertheless, the emergence of the structural equation model (SEM) framework of analysis recently facilitated modeling at multi levels and the inclusion of variations within and among individuals (Wickrama et al., 2016). Among such innovative analytical approaches is LGCM, which makes it possible to trace and examine alterations in human characteristics, such as personality traits, human behaviors, and academic achievement across time phases. One of the main properties of CDST is sensitivity to initial condition. This property is reflected in intercept factor of LGCM, which shows the initial level of a given construct like an L2 affective variable. The value of the intercept is compared with that of the slope indicting the growth of the construct.

Latent growth curve modeling has considerable methodological benefits for investigating panel data compared to the older regression analyses and comparison of mean scores. For example, the dynamic nature of the older types of models perceives the repeated measure as a mechanism that unfolds through time not as a fixed state at two distinctive phases of time (Coyne and Downey, 1991). If a single variation follows a trajectory marked by non-linearity, LGCM methods of analysis are pertinent to unraveling the complications of such variation (Willet and Sayer, 1994). Another advantage is that because of the developmental nature of the process of change, LGCM analytical framework gives researchers interested in empirical research the promises of a richer, wider and more in-depth array of research questions tapping on the quality of individual growth (Willet, 1988). Finally, the LGCM techniques offer a deep understanding of the dynamic relationship between a wider array of time-varying mental and non-mental constructs than the traditional analytical methods. The types of growth curve models used so far in language studies have been conventional LGM, parallel processing modeling (PPM), the curve of factors (CFM), and factor of curves (FCM; Wickrama et al., 2016).

Conventional growth curve is a two-stage process, one aiming to describe change through time for each individual (Willet and Sayer, 1994; Wickrama et al., 1997). And the other aims to estimate an average intercept, or initial state, (i.e., the mean of the variable of interest at the beginning of measurement) and an average slope, or the speed of variation, for all subjects, each with a unique variance (Wickrama et al., 2016). The main difference between LGCM and some classic models for the investigation of SLA variables across time such as repeated measures ANOVA is that in that first-order latent variables are involved in LGCM. Also, LGM is more flexible to missing data and unequal time intervals (see Curran et al., 2010). It should be noted that in case of evidence for nonlinear patterns of change in L2 affective variables, non-linear LGCM such as quadradic growth curve and piecewise growth curve models can be used.

Latent growth curve modeling with a focus on the analysis of change of only one variable is called univariate LGCM. LGCM has the potential to incorporate the parallel development two L2 affective variables. This model is referred to as a parallel process or bivariate LGCM. Parallel-process, or associated growth curve, provides a clearer and more sophisticated knowledge of the dynamic relationship between time-reliant socio-contextual variables and individual constructs than conventional analytical methods (Wickrama et al., 2016). LGCM can be extended to different models such as curve of factors and factor of curves models in which second-order factors of the constructs of interest are involved in the model. Factor curves modeling (FCM) diverges from the conventional LGCM uses composite measures and calculates a smaller number of parameters. Instead, FCM accounts for the distinctive role of the primary growth factors (level and slope of the construct) to the second-order factors within the model (initial condition and slope of variation in the construct). It is named a factor-of-curves model as it initially does the growth curve estimations and after that moves on to the definition of the factors of such curves (Duncan and Duncan, 1996). Higher-order modeling frameworks, including FCM, account for the different fluctuation of divergent components of a construct through time by making up primary growth factors that are exclusive to the dynamicity of each component. Confirmatory factor analysis (CFA) is more often employed to substantiate a foregoing proposition about the structure of a global factor that represents a behavior, construct or an internal variable. It reveals how a number of indicators (items) that evaluate specific sub-domains load on the global factor (known as the latent variable). The global factor of an L2 affective variable in a factor of curves model indicates to what extent the global factor can estimate the variance of the second-order intercept and slope.

The validity of a construct cross time is checked *via* longitudinal confirmatory factor analysis. Since longitudinal CFA is a prior assumption of LGCM, it should be seen as an inseparable process of the model. If a simple CFA is developed into a longitudinal CFA (LCFA) which repeatedly measures the trait through time, it helps to measure the invariance of the factor through time. It allows for testing whether the items of the latent factor adequately load on the factors of the single phenomenon of interest in a similar fashion through the passage of time (Wickrama et al., 2016; Hiver and Al-Hoorie, 2019). The time interval and the number of time points considered for conducting an LGCM of L2 affective variables depends on the study purpose and the construct *per se*. What follows is a review of the existing body of literature in L2 studies that have employed the LGCM approach in research methodology. The findings and implications for the field will show why it is worth waiting to see many more academic publications report findings of empirical studies using the LGCM techniques.

## Review of LGCM in L2 education

How L2 affective variables develop across time and how their growth level can be accounted by their initial level need to be deeply investigated. The studies in L2 education domain which have used the LGCM are limited in number, and they have been all conducted within the past few years. Yet, they had a significant contribution to the field, as will be reviewed here. A summary of these published works of research are included in Table 1.

**Table 1:** A summary of the related literature on latent growth curve modeling in language studies.

Title of paper	Authors (year)	Journal	Purpose of study	Main findings
Boredom in practical English language classes: a longitudinal confirmatory factor analysis-curve of factors model	Elahi Shirvan et al. (2021d)	Applied Linguistics Review	To investigate time-dependent variation of boredom in English Classes; to substantiate the longitudinal validity of the boredom in English classes-scale	Longitudinal validity of boredom scale was confirmed; EFL learners experienced a decreasing trend over time; and learners with higher initial states of boredom underwent a faster reduction of the trait through time
A longitudinal study of the subdomains of boredom in practical English language classes in an online setting: a factor of curves latent growth modeling	Kruk et al. (2021)	Journal of Multilingual and Multicultural Development	To examine the co-development of the subdomains of boredom in English language classes in an online setting over time	A statistically significant decrease was found over time in both subdomains of boredom; a negative covariance was found between initial state and growth level of each component; covariances between the initial and growth states of components were averagely high; and variances of the initial and growth conditions of every subdomain were explained by the global factor of boredom
Joint growth trajectories of trait emotional intelligence subdomains among l2 language learners: estimating a second-order factor-of-curves model with emotion perception	Taherian et al. (2021)	Frontiers in Psychology	To examine the developmental dynamics of trait emotional intelligence and its subdomains in EFL learning in a longitudinal work of research	Sufficient inter-individual changes and intra-individual tendencies within each component and a significant rise over time across the components; dynamicity of TEI proven during learning an L2
L2 grit: a longitudinal confirmatory factor analysis-curve of factors model	Elahi Shirvan et al. (2021a)	Studies in Second Language Acquisition	To examine temporal change of L2 grit in English classes; to examine the longitudinal validity of L2 grit scale; to expand the domain-specific stage of investigating L2 grit to dynamic phase	Longitudinal validity of L2 grit scale was confirmed; a steeper increase of L2 grit was found over time for learners with lower initial grit scores
Perseverance of effort and consistency of interest: a longitudinal perspective	Wang et al. (2021b)	Frontiers in Psychology	To trace the co-development of EFL learners’ perseverance of effort and consistency of interest, the two subdomains of grit, over a course time	There was evidence for inter-individual variation; covariance between intercept and slope of every component was investigated and for both the findings showed a negative covariance of the slope and intercept; covariance of intercepts and slopes of the two components was significantly positive; and variance of intercept and slope of each component showed to be explained by the global factor.
Foreign language enjoyment: a longitudinal confirmatory factor analysis–curve of factors model	Elahi Shirvan et al. (2021b)	Journal of Multilingual and Multicultural Development	To verify the validity of the innovative FLE scale	Model fit was accepted, invariance of latent factor was proven through the passage of time; negative covariance of slope and intercept suggested students of less initial FLE underwent a more rapid increase in FLE through the passage of time.
A longitudinal study of the subdomains of foreign language enjoyment: a factor of curves latent growth modeling	Taherian et al. (2021)	International Journal of Complexity in Education	To trace the co- development of English language learners’ private and social foreign language enjoyment (FLE)	All growth parameters across the two primary growth curves (intercepts and slopes of private and social FLE) were statistically significant; Existence of sufficient inter-individual variation and intra-individual trend within each component was confirmed.
A longitudinal study of foreign language enjoyment and L2 grit: A latent growth curve modeling.	Elahi Shirvan et al. (2021c)	Frontiers in Psychology	To trace the development of foreign language enjoyment (FLE) and L2 grit through time	The findings showed a rising trend in the correlation between the growth states of the two variables; covariances between FLE and L2 grit intercepts and slopes were statistically significant; the presence of a parallel process of FLE and foreign language grit was proven.
Change over time in learners’ mindsets about learning a foreign language	Elahi Shirvan et al. (2022)	Polish Psychological Bulletin	To trace changes in language mindsets over time *via* a curve of factors model	The model fit was confirmed; invariance of latent factor was proven over time; a negative covariance was found between the initial level language mindsets and the growth level; and students with a highly initial state of language mindsets underwent lower change in the variable through time.

In an innovative work of research into learner psychology, Elahi Shirvan et al. (2021d) firstly drew attention to the dynamic turn in SLA research. Secondly, they contended that learner-related psychological constructs such as foreign language learning boredom should be assessed from a longitudinal perspective. Then, they emphasized the need for expanding the application of measurement methods that can reflect the developmental features and temporal variations of language leaners’ boredom. The technique they suggested and actually used in their research was longitudinal confirmatory factor analysis-curve of factors model (LCFA-CFM), which was able to capture the nuanced changes of language learners’ boredom during an L2 course. This analytical method was dependent on the initial condition of this construct (boredom) and also its slope (rate or speed) of changes. In this study, Elahi Shirvan et al. (2021d) also hoped to measure the longitudinal validity of the boredom in English classes-revised (BPELC-R) measurement instrument developed by Pawlak et al. (2020a). The measurement instrument is an extended edition of a similar scale employed by Kruk and Zawodniak (2017), based on the Boredom Proneness Scale (BPS, Farmer and Sundberg, 1986).

The benefits of LCFA-CFM are that it ensures measurement invariance of the main variable (e.g., boredom) through the passage of time, takes into account the second-order latent variables, and involves the measurement of inter-personal variations while feeling the emotion (Elahi Shirvan et al., 2021d). The researchers collected their data using 412 adult EFL learners in four points of measurement using BPELC-R. Then, they analyzed the data in Mplus *via* LCFA-CFM. The authors contended that without using LCFA of BPELC-R, any observed change of boredom during the language learning process can be misunderstood. Evidence was also found for the different rate of changes in boredom among individual language learners. Moreover, the negative correlation between the intercept and slope of the participants’ boredom showed that students with higher initial states of boredom underwent a faster reduction of boredom through time.

Investigation of boredom in language learning from a dynamic perspective continued in another study by Kruk et al. (2021). This research was inspired by the need to explore language learners’ emotions, especially the under-researched emotions such as boredom, in online education. The theoretical framework was CDST. In this study, Kruk et al. (2021) used FCM to trace the mutual growth of the components of boredom in practical EFL courses in an online educational context through time in four stages of an online EFL program. The aim was to examine the covariance of the initial state and the slope (of change) of the components, and also to see the degree to which the changes in these components could be explained by the underlying global factor. To this aim, the researchers collected the data from 412 adult English language learners in stages of time using the Boredom in Practical English Classes – Revised scale, developed by Pawlak et al. (2020a). The Mplus was employed for the statistical analyses conducted in three levels. The results indicated a statistically significant reduction through time in both components of boredom. Moreover, the analysis revealed a negative covariance of the initial state and the growth state of each component of boredom. Besides, the covariances of the initial and growth states of both components were to some extent high. Also, as the results revealed, the variances of the initial and growth states of each component were explained largely by the latent global factor of boredom (in language learning).

Another language learner emotion that was studied longitudinally and dynamically through the LGCM techniques has been emotional intelligence. Taherian et al. (2021) traced the dynamic nature of trait emotional intelligence (TEI) and its components over an EFL learning course through longitudinal research. To this aim, they recruited 309 EFL learners (of both sexes) to trace the trajectories of TEI as the latent factor and the parallel growth of the TEI components for 1 year in classroom-based language learning using PPM and FCM. In addition, emotion perception (EP) was employed as a distal outcome to explore how growth parameters (i.e., the intercept and slope) in a TEI-FCM affect EP distal outcome. The FCM analysis showed adequate interpersonal variation and intrapersonal trajectories of change in each component of the trait and also showed a significant rise through time among the four components of the trait. As for the covariances within and between the components of TEI, the PPM findings showed moderate-to-high correlations between the intercept and slope growth factors within and among the components.

Concerning the direct correlation between the global growth factors (intercept and slope) of TEI and EP, the findings showed that the global TEI intercept and slope were significantly correlated with EP. In particular, the intercepts and slopes of emotionality and sociability showed to be strongly correlated with EP. These findings point to the developmental nature of TEI while learning an L2 as elaborated on by Taherian et al. (2021) in relation to the potential factors related to TEI and the existing body of literature.

Among the L2 learner related factors, grit and its two components (i.e., perseverance of efforts and consistency of interest) were also investigated dynamically through the LGCM techniques. Elahi Shirvan et al. (2021a) drew attention to the longitudinal quality of L2 grit and the inefficiency of traditional research methods using cross-sectional designs to prove the validity of foreign language grit scale. Thus, they conducted their research to expand the domain-specific stage of L2 grit research, with an emphasis on long-term goals, into a dynamic stage. To this aim, they used an LCFA-CFM approach to assess variation in L2 learners’ grit at different phases of time during an EFL program. Elahi Shirvan et al. (2021a) acknowledged the benefits of the LCFA-CFM approach, as it ensured measurement invariance through time, dealt with second-order latent variables, took into account the errors in measurement, and were able to assess interindividual variation. Thus, they first used the LCFA to examine the factor invariance of L2 grit according to a bi-factorial CFA model through the passage of time. Then, they used CFM to trace variations within L2 grit over an EFL course. They collected the required data from 437 EFL adult learners in Iran in four stages of time *via* the L2 grit scale and used Mplus 7.4 for data analysis. As the results showed, the model fit was confirmed and the invariance of the L2 grit latent factor was proven through time. Furthermore, the negative covariance between the initial state of L2 grit and its slope of variation through time (second-order latent constructs) showed a faster rise in the L2 grit through time for language learners with lower scores of this factor at the beginning of measurement. In other words, language learners who began at a higher L2 grit underwent fewer changes in L2 grit through the passage of time. The LCFA-CFM tried to prove that the L2 grit factor structure was stable (not changing) through the passage of time and offered insights into how L2 grit can vary within an EFL course.

The dynamic line of research into L2 grit continued with longitudinal research conducted by Wang et al. (2021b) on language learners’ perseverance of efforts (PE) and consistency of interest (CI). This research was also enriched by the dynamic view of language education, and intended to assess the parallel growth of EFL learners’ two dimensions of L2 grit (i.e., PE and CI) over a flow of time. To this aim, Wang et al. (2021a) used an FCM technique to examine the covariance of the components of L2 grit longitudinally through the passage of time, to see how alterations in PE and CI are influenced by their initial levels; and the degree to which the changes in the two subdomains/components are explained by the latent L2 grit construct. To collect the data, the L2 grit scale was completed by 1,384 EFL adult learners in four stages of assessment, and Mplus was used to analyze the data using FCM in three stages. In exploring the direction of variation in PE and CI, the results revealed that a higher mean was calculated in the growth (the slope) than the intercept. The analysis also revealed interpersonal variation. Furthermore, the covariance of the intercept and slope of each component was assessed and for both (i.e., PE and CI) the results suggested a negative covariance between the slope and intercept. The covariance of the intercepts and the slopes of the two components were significant and positive. The variance of the intercept and slope of every component showed to be largely explained by the latent global factor (i.e., L2 grit).

Besides language learners’ boredom and grit, foreign language enjoyment (FLE) construct was also investigated longitudinally from a CDST perspective using the LGCM techniques of analysis. Elahi Shirvan et al. (2021b) adopted a LCFA-CFM technique to test and confirm the validity of the basic FLE scale (Dewaele and Mac Intyre, 2014) longitudinally and document variations in this variable in different phases of measurement in an EFL course. Elahi Shirvan et al. (2021b) contended that this analytical approach ensured invariance in measurement through time and accounted for second-order latent variables. The data were gathered from 437 adult English language learners in four stages of time using FLE scale and were analyzed in Mplus using LCFA-CFM. The results showed that the model fit was confirmed and the latent factor invariance was proven through time. The negative covariance of slope and intercept (second order latent variables) showed that students with lower initial FLE went under a more rapid rise in FLE through time, because the mean of slope was larger than that of the intercept, which can be dependent on L2 learner’s motivation, different attitude to language learning and the teacher’s role to support students. The researchers reckoned that the main strength of their research was the longitudinal confirmatory factor analysis used to trace the temporal development of the construct and how it grew during the EFL learning program.

The two dimensions of FLE (i.e., private and social FLE) were also explored from a dynamic approach by to record the parallel growth of EFL learners’ FLE. To this aim, an FCM was used, as an innovative statistical method, to document the covariance of the components of FLE longitudinally on four occasions of measurement and to examine the covariance of the initial state and slope of the FLE components and how variation in private and social FLE can be explained by the latent FLE global factor. To collect the required data, the FLE scale created by Dewaele and Mac Intyre (2014) was provided for 437 adult English language learners in four stages of time. Mplus was used for data analysis within three steps as recommended by Wickrama et al. (2016). The findings showed that all growth parameters across the two primary growth curves (private FLE and social FLE intercepts and slopes) were statistically significant. This finding approved the presence of enough inter-individual and intra-individual variation. Also, the tendency within each component of FLE enjoyed a significant increase through time in these components. The primary growth factors of private and social FLE contributed differentially to FLE second-order growth factors. At the end, the intercept and slope variances of each second component showed to be primarily accounted for by the FLE global factor.

The LGCM techniques make it possible to trace the development and variation between more than one latent variable too. For example, Elahi Shirvan et al. (2021c) emphasized the need for developing appropriate methods to examine the nuanced dynamic features of emerging constructs in l2 education research including grit and enjoyment. They aimed to explore the development of FLE and L2 grit through time. To this aim, they used a bivariate LGCM to assess the covariance between 437 English language learners’ initial and growth states of L2 grit and FLE across four points of time with 2-week intervals. The original FLE scale and the L2 grit scale were employed to collect the data. The model which represented the covariance between intercepts and slopes of FLE and L2 grit was tested in Mplus 7. The results showed a rising rate in the correlation between the growth states of the two variables. In other words, at their growth level, the mean scores of both L2 grit and FLE were larger than the initial level. The further analysis of the co-variations within the model showed that those between the FLE and L2 grit intercepts and slopes were statistically significant. That was an evidence for the presence of a parallel process (co-development) of the two constructs. This finding also meant that a rise in the FLE level among the language learners was correlated significantly with a rise in L2 grit during the whole language learning program.

Another learner related variable that was explored dynamically with the LGCM techniques in recent years is mindset. Influenced by the recent lines of longitudinal research in L2 education, Elahi Shirvan et al. (2022) traced changes in language mindsets through time using a CFA analytic technique. To collect the data, 437 adult EFL learners completed the Language Mindsets Index in four points of measurement. The results showed that the model fit was confirmed and the latent factor invariance was proven through the passage of time. The results pointed to a negative correlation between the initial state of language mindsets and the growth state of the variable. It shows that language learners with a higher initial state of language mindsets underwent fewer variation in the mindset through the passage of time, yet research subjects with a lower level of language mindset altered their mindsets more through the passage of time. The pedagogical benefits of the results included language teachers’ attention to the usefulness of growth language mindset interventions.

## Insights for prospective line of research

Despite being a variable-centered method, LGCM measures both change, stability, and sensitivity to initial conditions in the investigation of L2 affective variables. The temporal change in the L2 affective variables indicate that these variables should not be seen as traits as they might undergo change in different time occasions. In the light of the increasing line of research with a dynamic approach, there is a considerable need for methods of estimation that manage to consider intra-individual variations and inter-individual differences. The LGCM techniques used in the above-mentioned studies enabled the researchers to focus on within-individual changes, within-subdomains trajectories and the differences between the subdomain growth factors of the L2 learner related variables (e.g., boredom, enjoyment, grit, trait emotional intelligence, and mindset), resulting in more accurate and nuanced conclusions concerning the global factors. The share of contribution of each primary growth factor to the variation of the global factor could be specified. Contrary to composite measures, which are computed assuming equal contributions (weights) of different subdomains, the LGCM techniques (especially FCM) allow the components of a given construct to behave differently through time by accounting for primary growth factors specific to each component.

The future line of L2 education research needs to essentially explore L2 learner related variables longitudinally to reflect a more realistic, dynamic conceptualization of the variable of interest as it grows naturally in the fully interactive classroom context. Accordingly, it is expected that the measurement represents this dynamic shift. The statistical model adopted should represent the co-development of the components of the trait and take into account the initial state and also the growth of variation in the components. It is supposed to consider inter-individual differences and the effect of the global latent variable on changes in the growth of primary components. Traditional statistical procedures are incapable of meeting these requirements and, therefore, only give us a static image of the variable of interest, which fails to represent its growing quality in the ecology of classroom. Old methods often take mean groups scores to represent subjects’ achievement or a specific psychological or personality-related construct, and lose sight of inter-individual differences that naturally characterize the growth of human qualities or behaviors.

The LGCM techniques employed in the aforementioned studies can be used as efficiently to assess other human traits in language learner (or possibly teacher) psychology, such as well-being, compassion, mindful attention/awareness, loneliness, and so on. In the dynamic context of a language classroom, characterized by the interactive nature of a multitude of personal (teacher or learner) and contextual variables, the LGCM techniques can be effectively applied in tracing the development of the constructs in the time-dependent phases of the L2 program nested within the dynamic context of classroom learning. LGCM seems to meet the requirements of measuring multifaceted human traits from a CDST perspective and can, therefore, adequately and promisingly contribute to the existing related literature in L2 education, with a long-held interest in L2 learners’ personality traits including motivation, mindset, attitude, and passion for learning. LGCM can be applied to teacher-student interpersonal factors (for a comprehensive review, see Xie and Derakhshan, 2021) as well as the factors embodied in positive psychology in L2 education (Wang et al., 2021a).

## Conclusion

The present review cautions against the issues with conventional statistical frameworks to delve into multi-faceted human learners’ traits such as boredom, grit, emotional intelligence, and the like. It goes on to emphasize the need to avoid a simplistic integrative or holistic attitude to any language learner related construct and, instead, highlights the essentiality of tracing the trajectory of changes in the components of variables, checking for the covariances, and accounting for intra-and inter-individual variation. The present study draws attention to the insufficiencies of the mere reliance on single-shot designs of study and the unrealistic static conceptualization of L2 learners’ variables which can result in inefficient decision-making about individual L2 learners in foreign language education programs. As contended by Curran and Willoughby (2003), LGCM techniques combine features of the nomothetic and idiographic approach and thus make it possible to take advantage of both procedures to draw more realistic conclusions from applied linguists’ data. LGCM is capable of modeling a unique trajectory for each subject, a mean trajectory of all subjects, along with the variability about this mean (Hiver and Al-Hoorie, 2019). As the studies reviewed in this paper show, there is a noticeable lacking use of LGCM in L2 education studies. Many L2 learner or teacher-related variables are left unattended from a dynamic approach. Appropriate quantitative and qualitative research methods are, thus, deemed essential to embrace the complex city and dynamicity involved in language learning. Accordingly, LGCM techniques are highly recommended to enrich the empirical works of research in L2 education. It is estimated that with the recent application of LGCM in the investigation of L2 affective variables, the expansions of LGCM can be the focus of future research on these variables.

## Author contributions

The author confirms being the sole contributor of this work and has approved it for publication.

## Funding

This study was supported by the Anhui Province Education & Teaching Research and Reform Project administered by the Education Department of Anhui Province in 2020-A study on the Cultivation Model of College English Cross-cultural Competence in Science & Engineering Universities (No: 2020jyxm0471).

## Conflict of interest

The author declares that the research was conducted in the absence of any commercial or financial relationships that could be construed as a potential conflict of interest.

## Publisher’s note

All claims expressed in this article are solely those of the authors and do not necessarily represent those of their affiliated organizations, or those of the publisher, the editors and the reviewers. Any product that may be evaluated in this article, or claim that may be made by its manufacturer, is not guaranteed or endorsed by the publisher.
